# Maternal plasma lipid levels across pregnancy and the risks of small-for-gestational age and low birth weight: a cohort study from rural Gambia

**DOI:** 10.1186/s12884-020-2834-1

**Published:** 2020-03-12

**Authors:** Sandra G. Okala, Ebrima A. Sise, Fatou Sosseh, Andrew M. Prentice, Laura A. Woollett, Sophie E. Moore

**Affiliations:** 1 Department of Women and Children’s Health, King’s College London, London, SE1 7EH UK; 2 Department of Pathology and Laboratory Medicine, University of Cincinnati, Cincinnati, Ohio USA; 3 MRC Unit The Gambia at the London School of Hygiene and Tropical Medicine, Banjul, The Gambia

**Keywords:** Maternal lipids, Cholesterol, Triglycerides, Birth weight, Low birth weight, Small-for-gestational-age, Pregnancy, The Gambia

## Abstract

**Background:**

Sub-optimal maternal lipid levels during pregnancy may be implicated in the pathophysiological mechanisms leading to low birth weight (LBW) and small-for-gestational-age (SGA). We aimed to determine whether maternal lipid levels across pregnancy were associated with birth weight and the risks of LBW and SGA in rural Gambia.

**Methods:**

This secondary analysis of the ENID trial involved 573 pregnant women with term deliveries. Plasma levels of total cholesterol (TC), high-density lipoprotein cholesterol (HDL-c), low-density lipoprotein cholesterol (LDL-c), and triglycerides (TG) were analyzed at enrolment (mean (SD) = 13.9 (3.3) weeks gestation), 20 and 30 weeks gestation as continuous variables and percentile groups. Regression models with adjustment for confounders were used to examine associations between gestational lipid levels and birth weight and the risks of LBW (birth weight < 2500 g) and SGA (<10th percentile INTERGROWTH-21ST for birth weight).

**Results:**

There were 7.9% LBW and 32.5% SGA infants. At enrolment, every unit increase in HDL-c was associated with a 2.7% (*P* = 0.011) reduction in relative risk of LBW. At 20 weeks gestation, every unit increase in TC levels was associated with a 1.3% reduction in relative risk of LBW (*P* = 0.002). Low (<10th percentile) HDL-c at enrolment or at 20 weeks gestation was associated with a 2.6 (*P* = 0.007) and 3.0 (*P* = 0.003) times greater risk of LBW, respectively, compared with referent (10th─90th) HDL-c. High (>90th percentile) LDL-c at 30 weeks gestation was associated with a 55% lower risk of SGA compared with referent LDL-c (*P* = 0.017). Increased levels of TC (β = 1.3, *P* = 0.027) at 20 weeks gestation and of TC (β = 1.2, *P* = 0.006) and LDL-c (β = 1.5, *P* = 0.002) at 30 weeks gestation were all associated with higher birth weight.

**Conclusions:**

In rural Gambia, lipid levels during pregnancy were associated with infant birth weight and the risks of LBW and SGA. Associations varied by lipid class and changed across pregnancy, indicating an adaptive process by which maternal lipids may influence fetal growth and birth outcomes.

**Trial registration:**

This trial was registered as ISRCTN49285450 on: 12/11/2009.

## Background

Birth weight is a retrospective indicator of fetal growth and maternal health during pregnancy, but also a subsequent predictor of long-term health outcomes for both the mother and her offspring [1–4]. Low birth weight (LBW) is defined as a birth weight below 2500 g and small-for-gestational-age (SGA) as a birth weight less than the 10th percentile for the gestational age [1, 4–6]. In 2015, 20.5 million infants were born LBW, representing 14.6% of all births [7]. Most (91%) LBW infants were born in low- and middle-income countries (LMICs) with almost three-quarters in Asia and sub-Saharan Africa [7]. It has been estimated that in 2012, one in five infants from LMICs were born SGA, representing 23.3 million births in that year [8]. Both LBW and SGA are important indicators used to identify infants at greater risks of morbidity and mortality [4]. LBW infants are about 20 times more likely to die within the neonatal period compared to those born with a normal birth weight (NBW; ≥2500 g) and as a result, LBW is implicated in 60 to 80% of all neonatal deaths [4, 9]. In a study conducted in Mozambique (*n =* 5542), infants born SGA were found to have five times higher rates of mortality compared to adequate-for-gestational-age (AGA) infants [6].

Accumulating evidence indicates that imbalanced lipid levels during pregnancy may alter fetal lipid metabolism, thereby impacting fetal growth and birth weight, and the metabolism of both the mother and her offspring [10–12]. During pregnancy, pronounced changes in lipid metabolism occur characterized by an elevation of maternal lipids to support the physiological adaptation to gestation and the nutritional and hormonal needs of the gestating mother and the growing fetus [13]. Maternal malnutrition, inflammation or infection during pregnancy may lead to an inadequate response to pregnancy-induced changes in lipid metabolism, abnormal maternal and fetal lipid levels and adverse birth outcomes.

Previous investigations examining potential associations between maternal lipid levels and birth weight have mostly focused on the second trimester of pregnancy and have often used lipid measurements from a single gestational time-point. When compared to mid-range values, low total cholesterol levels (TC) have been associated with lower birth weight [12, 14] and greater risk of LBW [15], and high TC [12, 16] or high triglycerides (TG) levels with higher birth weight [12, 15–19]. In a case-control study conducted in Canada, higher mean levels of high-density lipoprotein (HDL) particles in the second trimester were found in women with term-born SGA infants compared to women with AGA infants [20]. In a population-based study from China, high TG in the third trimester was found associated with reduced risk of SGA [10].

Although LMICs carry the highest burden of LBW and SGA cases, there is a paucity of data on the influence of maternal gestational lipids on birth weight-related outcomes in these settings which may hamper the development of appropriate preventive, screening and prophylactic interventions. The current study aimed to examine associations between maternal lipid levels across pregnancy with birth weight and the risk of LBW and SGA in a cohort from a food-insecure region of rural Gambia, in sub-Saharan Africa.

## Methods

### Study population

**Fig. 1:**
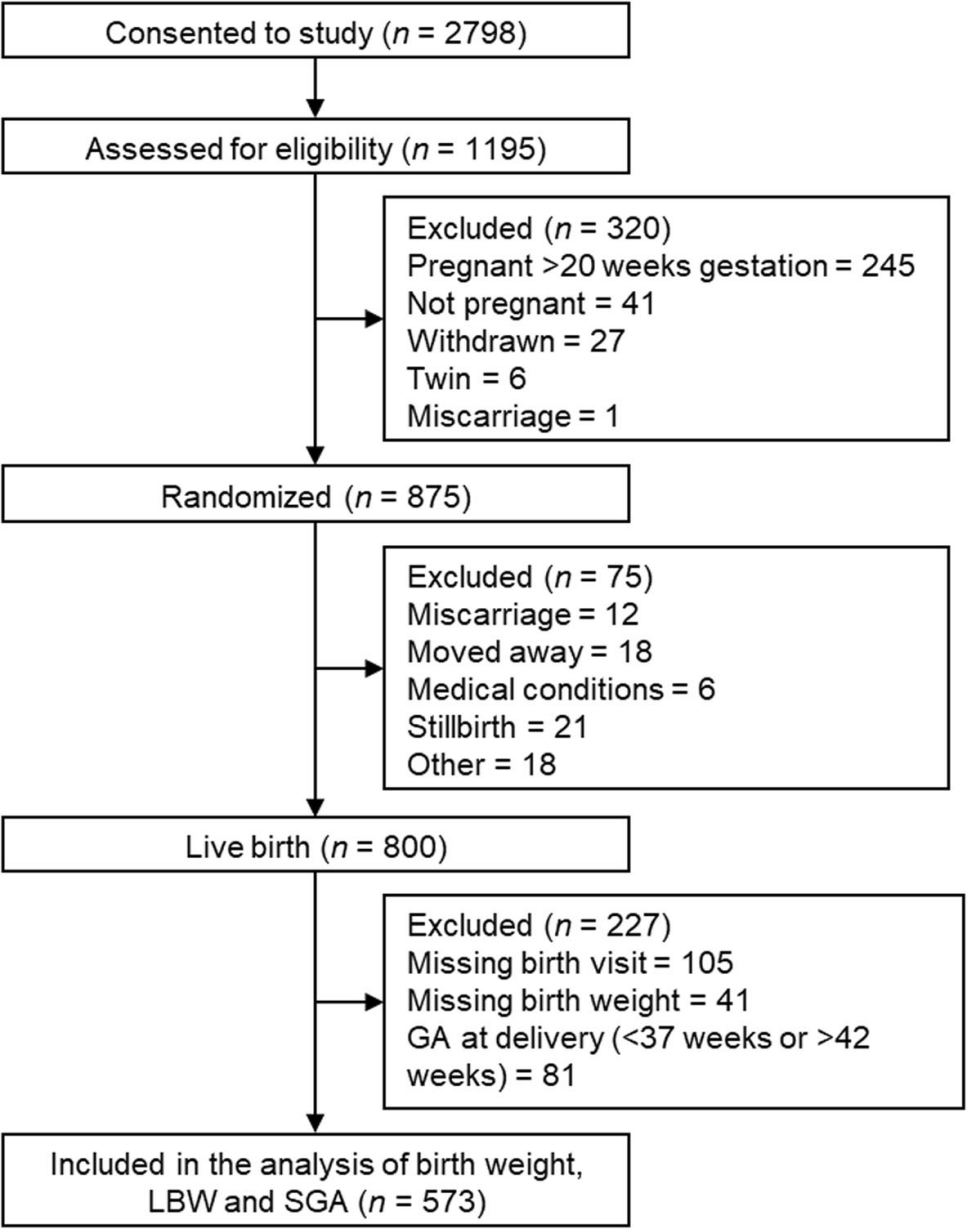
Flow chart of trial participants included in the analysis Abbreviations: LBW; low birth weight and SGA; small-for-gestational-age

This study is a secondary analysis of the Early Nutrition and Immune Development (ENID) trial; ISRCTN49285450]), a randomized, partially blinded trial investigating the impact of prenatal and infancy nutritional supplementation on infant development in the West Kiang region of The Gambia. The published ENID trial protocol provides complete details of the trial [21] while an overview of relevant information to the current secondary analysis is included here (participant selection outlined in Fig. 1). All non-pregnant women aged 18 to 45 years registered in the West Kiang Demographic Surveillance System (DSS) were invited to participate in the trial [22]. After written consent, all women were visited monthly. Between January 2010 and June 2013, 2798 women were recruited for monthly surveillance of pregnancy. Women who missed their last menses and had a positive urine pregnancy test were invited to the Medical Research Council (MRC) Keneba clinic for an ultrasound examination of pregnancy status and stage. Of the 1195 women with a positive pregnancy test, those confirmed as pregnant but with a gestational age ≥ 20 weeks or a multiple pregnancy or those confirmed as HIV positive or with severe anemia (hemoglobin (Hb) < 7 g/dL) were excluded from the study. A total of 875 pregnant women who met the inclusion criteria were randomized into the antenatal supplementation phase of the trial, yielding 800 live births. For the current analysis, 227 infants were excluded due to missing birth visit (*n =* 105) or birth weight (*n* = 41) or not born at term with gestational age at delivery below 37 weeks or over 42 weeks (*n* = 81), resulting in 573 mother-newborn pairs (Fig. 1). Of note, preterm infants were not included in this analysis due the low numbers of preterm births within the ENID cohort (*n* = 14).

### Intervention and procedures

The randomization procedure allocated eligible pregnant women to one of four prenatal nutritional supplements; (i) iron folic-acid (FeFol) tablets, the standard of care as per Gambian Government guidelines; (ii) multiple micronutrients (MMN) tablets, a combination of 22 micronutrients designed for use during pregnancy by UNICEF/WHO/UNU [23]; (iii) protein-energy and iron-folate (PE + FeFol), a lipid-based nutritional supplement (LNS); and (iv) protein-energy and multiple micronutrients (PE + MMN). Supplement composition is described in Additional Table A1. Field staff visited enrolled women weekly for supplement provision. During weekly visits, compliance to supplement was evaluated by an assessment of the quantity of supplements not used, and a record of maternal morbidity was also collected.

At enrolment (mean gestational (SD) = 13.9 (3.3) weeks gestation), gestational age was assessed using a Siemens ACUSON Antares Ultrasound Imaging System (Siemens Medical Solutions USA, Inc., California, USA) with a CH6–2 (5.71 MHz) transducer). Further clinic visits were conducted at 20 and 30 weeks gestation. At all visits, data were collected on maternal anthropometry, Hb, blood pressure, and a urinary analysis was performed. A sample of venous blood (10 mL) was also collected following an overnight fast and plasma samples were stored on ice until processing within 1 h of collection. Standardized and validated equipment and standard operating protocols were used and applied for all measurements. A study midwife visited women and their newborns within 72 h of delivery for a standard maternal and newborn health check. Infant weight and length were measured using digital infant scales (Seca mobile digital baby-scale 334; UK) with 10 g precision and a portable infant roll meter (Rollameter 100; Harlow Healthcare, UK) to the nearest 0.1 cm, respectively.

### Laboratory analysis of maternal lipids

All maternal plasma samples were collected after an overnight fast and analyzed in the MRC Keneba laboratory for TC, HDL-c, low-density lipoprotein cholesterol (LDL-c), and TG levels using enzymatic colorimetric assays with Roche/Hitachi reagents and COBAS INTEGRA® 400 plus analyzer (Roche Diagnostics, Indianapolis, IN). The few samples that were physiologically aberrant, such as samples with detectable HDL-c and LDL-c levels but with a TC level near zero, were excluded from the analysis.

### Outcomes and cofactors

Low birth weight (LBW) was characterized as a birth weight below 2500 g and compared to normal birth weight (NBW) (≥2500 g) [9]. Small-for-gestational-age (SGA) was defined as a birth weight-for-gestational-age below the 10th percentile of the INTERGROWTH-21ST standards for birth weight in comparison to adequate-for-gestational (AGA) (≥10th percentile of the INTERGROWTH-21ST standards for birth weight) [24]. The West Kiang DSS was used to verify mother's birth date and age at enrolment [22]. A questionnaire collected at enrolment was used to determine maternal parity (i.e. numbers of stillbirths and live births). School attendance was defined as a binary variable (yes/no) based on whether enrolled women reported 1 year or more of English or Arabic school and not as the number of school years attended due to the low attendance mean (SD) (0.31 (0.62) years). Maternal BMI was calculated as weight (kg)/height (m)^2^ at each measurement time point in gestation. Maternal gestational weight gain was calculated by subtracting maternal weight between; enrolment and 20 weeks gestation, 20 and 30 weeks’ gestation, and enrolment and 30 weeks gestation, and by dividing each subtraction by the number of weeks between weight measurements and expressed in kg/week. Maternal morbidity was determined based on the number of self-reports of morbidity episodes (e.g. fever, nausea/vomiting, dysuria, bleeding and abdominal pain) assessed by questionnaire and divided by the number of weeks enrolled in the trial (n/week). Compliance to supplementation from enrolment to delivery (%) was computed by dividing the number of jars of LNS products (PE and PE + MMN) (empty, half-empty, and full) or count of tablets (MMN and FeFol) the women consumed by the number received and multiplying by 100. Birth season of the infant was defined as dry (November to May) or rainy (June to October).

### Statistical analyses

Maternal and infant variables were compared between LBW and NBW infants, and between SGA and AGA infants by Student’s t-test with Welch correction for unequal sample sizes. Unadjusted mean TC, HDL-c, LDL-c, and TG levels at enrolment and 20 and 30 weeks’ gestation were calculated with 95% confidence intervals (95% CI) and compared by Student’s t-test. Changes in maternal lipid levels across gestation were examined using a Wilcoxon rank-sum test for ordered groups and a paired sample t-test. Maternal lipid levels were analyzed as continuous variables and grouped into low (<10th), referent (10th–90th), and high (>90th) percentiles. Binary regression models were used to measure the relative risks (RR) of LBW and SGA associated with maternal lipid levels included as continuous or categorical variables (by percentile groups). Reduction in relative risk (RRR) expressed as a percentage was calculated as RRR% = (1-RR) × 100. The RR values for the analyses of the risks of LBW and SGA are presented in Additional Tables A2 and A3, respectively. Linear regression models were used to investigate associations between maternal lipids and birth weight (g) as an outcome. Models were adjusted for confounding factors selected based on previous research and on whether they significantly impacted on the models. For LBW and birth weight outcomes these included enrolment maternal age, parity, supplement groups (FeFol, MMN, PE or PE + MMN), gestational age, BMI, Hb level at lipid measurements, and compliance to supplement during pregnancy and infant birth sex and birth season. For SGA, the same confounding factors were used, but excluding gestational age. A previous analysis of the ENID dataset has shown a complex relationship between season and gestational weight gain on birth outcomes [25]. Given the potential relevance of changes in maternal BMI over pregnancy on gestational lipid levels, we also fitted BMI and changes in BMI within the models presented here. Statistical analyses were conducted with Stata version 15 (StataCorp LP Texas, USA).

## Results

### Cohort characteristics

A total of 573 pregnant women with singleton term infants were included in this study. There were 45 (7.9%) cases of LBW and 186 (32.5%) cases of SGA. At enrolment, 20.6% of women were underweight (BMI < 18.5 kg/m^2^), 68.2% were normal weight (BMI 18.5─24.9), 9.1% were overweight (BMI 25─29.9) and 2.1% were obese (BMI ≥ 30 kg/m^2^). Underweight women were significantly more likely to have a LBW (12.7% vs 6.4%, *P* = 0.023) or SGA (41.5% vs 29.7%, *P* = 0.014) infant compared to women with a BMI > 18.5 kg/m^2^. Table 1 compares the descriptive characteristics of the participants, split according to LBW versus (vs) NBW and SGA vs AGA. Women with LBW infants were enrolled later in the trial, were more likely to be nulliparous, had lower gestational age at delivery, a lower BMI at all time points of gestation and a lower gestational weight gain between enrolment (mean gestational (SD) = 13.9 (3.3) weeks gestation) and 20 weeks gestation compared with women with NBW infants (all, *P* < 0.05). LBW infants were all born SGA and were more likely to be female (*P* = 0.012). Women with SGA infants also had a lower BMI at all gestational time points (all, *P* < 0.01), a lower gestational weight gain between enrolment and 30 weeks gestation (*P* = 0.028) and higher gestational age at delivery (*P* = 0.012) compared with women with AGA infants. SGA infants were more likely to be born with a normal birth weight than with a LBW (75.8% vs 24.2%, *P* < 0.001). There were no significant differences in risk of LBW or SGA by maternal nutritional supplement group.

**Table 1:** Comparison of participants characteristics (*n* = 573) by LBW and SGA infant status^a^

**Variables**^**b**^	**LBW (*n*** **= 45)**	**NBW (*n*** **= 528)**	***p-value***^***c***^	**SGA (*n*** **= 186)**	**AGA (*n*** **= 387)**	***p-value***^***c***^
	**Mean (SD)**	**Mean (SD)**		**Mean (SD)**	**Mean (SD)**	
**Maternal variables**						
**Enrolment**^**d**^						
Gestational age (weeks)	14.9 (3.4)	13.8 (3.29)	**0.034**	14.1 (2.9)	13.8 (3.5)	0.249
Age (years)	29.2 (7.3)	30.4 (6.5)	0.274	30.3 (6.7)	30.3 (6.5)	0.888
Parity (*n*)	3.5 (2.7)	4.3 (2.6)	0.072	4.1 (2.7)	4.3 (2.5)	0.504
Nulliparous, *n (%)*	9 (20.0)	40 (7.7)	**0.005**	39 (8.1)	10 (12.1)	0.242
Educated, *n (%)*	7 (15.9)	114 (22.0)	0.348	38 (20.8)	83 (21.8)	0.711
Hb (g/L)	11.4 (1.5)	11.3 (1.4)	0.823	11.3 (1.5)	11.4 (1.3)	0.619
Supplement group, *n (%)*			0.916^e^			0.323^e^
FeFol	10 (22.2)	123 (23.3)		48 (25.8)	85 (22.0)	
MMN	13 (28.9)	143 (27.1)		49 (26.3)	107 (27.7)	
PE	10 (22.2)	129 (24.4)		46 (24.7)	93 (24.0)	
PE + MMN	12 (26.7)	133 (25.2)		43 (23.1)	102 (26.4)	
BMI (kg/m^2^)	19.7 (3.1)	21.1 (3.4)	**0.008**	20.3 (3.0)	21.3 (3.5)	**0.001**
BMI group), *n (%)*			**0.032**^e^			**0.012**^e^
Underweight (< 18.5 kg/m^2^)	15 (34.1)	103 (19.6)		49 (26.8)	69 (17.9)	
Normal weight (18.5─24.9 kg/m^2^)	26 (59.1)	365 (69.5)		119 (65.0)	272 (70.5)	
Overweight (≥25 kg/m^2^)	3 (6.8)	57 (10.9)		15 (8.2)	45 (11.7)	
Weight gain (enrolment to 20 weeks) (kg/week)	0.28 (0.30)	0.38 (0.35)	**0.048**	0.34 (0.31)	0.39 (0.37)	0.062
**20 weeks gestation**						
Hb (g/dL)	10.7 (1.3)	10.9 (1.15)	0.435	10.8 (1.2)	10.9 (1.1)	0.382
BMI (kg/m^2^)	20.8 (3.0)	22.0 (3.2)	**0.010**	21.1 (2.8)	22.3 (3.3)	**< 0.001**
Weight gain (20 to 30 weeks) (kg/week)	0.30 (0.16)	0.30 (0.21)	0.986	0.29 (0.18)	0.31 (0.22)	0.256
**30 weeks gestation**						
Hb (g/dL)	10.7 (1.4)	10.6 (1.3)	0.501	10.7 (1.4)	10.6 (1.3)	0.320
BMI (kg/m^2^)	21.9 (3.0)	23.1 (3.1)	**0.014**	22.2 (2.7)	23.4 (3.2)	**< 0.001**
Weight gain (enrolment to 30 weeks) (kg/week)	0.30 (0.13)	0.33 (0.18)	0.260	0.30 (0.15)	0.34 (0.19)	**0.028**
**Across pregnancy**						
Morbidity episodes (*n*/week)	0.18 (0.25)	0.20 (0.24)	0.694	0.17 (0.19)	0.20 (0.26)	0.117
Compliance to nutritional supplement (%)^f^	87.6 (9.8)	87.3 (14.4)	0.839	88.0 (12.6)	87.0 (14.7)	0.393
Gestational age at delivery (weeks)	39.5 (1.1)	40.2 (0.97)	**< 0.001**	40.3 (1.0)	40.1 (0.97)	**0.012**
**Infant variables**						
Sex, *n (%)*: Female	31 (68.9)	261 (49.4)	**0.012**	92 (49.5)	200 (51.7)	0.619
Birth season, *n (%)*: dry (Nov-May)	33 (73.3)	322 (61.0)	0.101	122 (65.6)	233 (60.2)	0.214

^a^The total sample comprised 573 mother-infant pairs and was compared according to LBW (< 2.5 kg) versus NBW (≥2.5 kg) and SGA (<10th percentile INTERGROWTH-21ST for birth weight) vs AGA (≥10th percentile INTERGROWTH-21ST for birth weight) and each comparison included the total sample (n = 573)

^b^Values are means with standard deviation (SD) unless otherwise specified

^c^P-values were calculated from Student’s t-test with Welch correction for unequal sample sizes by comparing LBW to NBW groups and SGA to AGA groups

^d^Mean (SD) gestational age at enrolment was 13.9 (3.3) weeks gestation

^e^*P*-values were calculated by ANOVA

^f^Compliance to supplement was calculated by dividing the number of jars or tablets the women consumed by the number she received and multiplying by 100

*Abbreviations*: *AGA* adequate-for-gestational-age, *BMI* body mass index, *FeFo*l iron and folic acid, *Hb* hemoglobin, *LBW* low birth weight, *MMN* multiple micronutrient, *NBW* normal birth weight, *PE* protein energy, *SGA* small-for-gestational-age

### Changes in maternal lipid levels across pregnancy

**Fig. 2:**
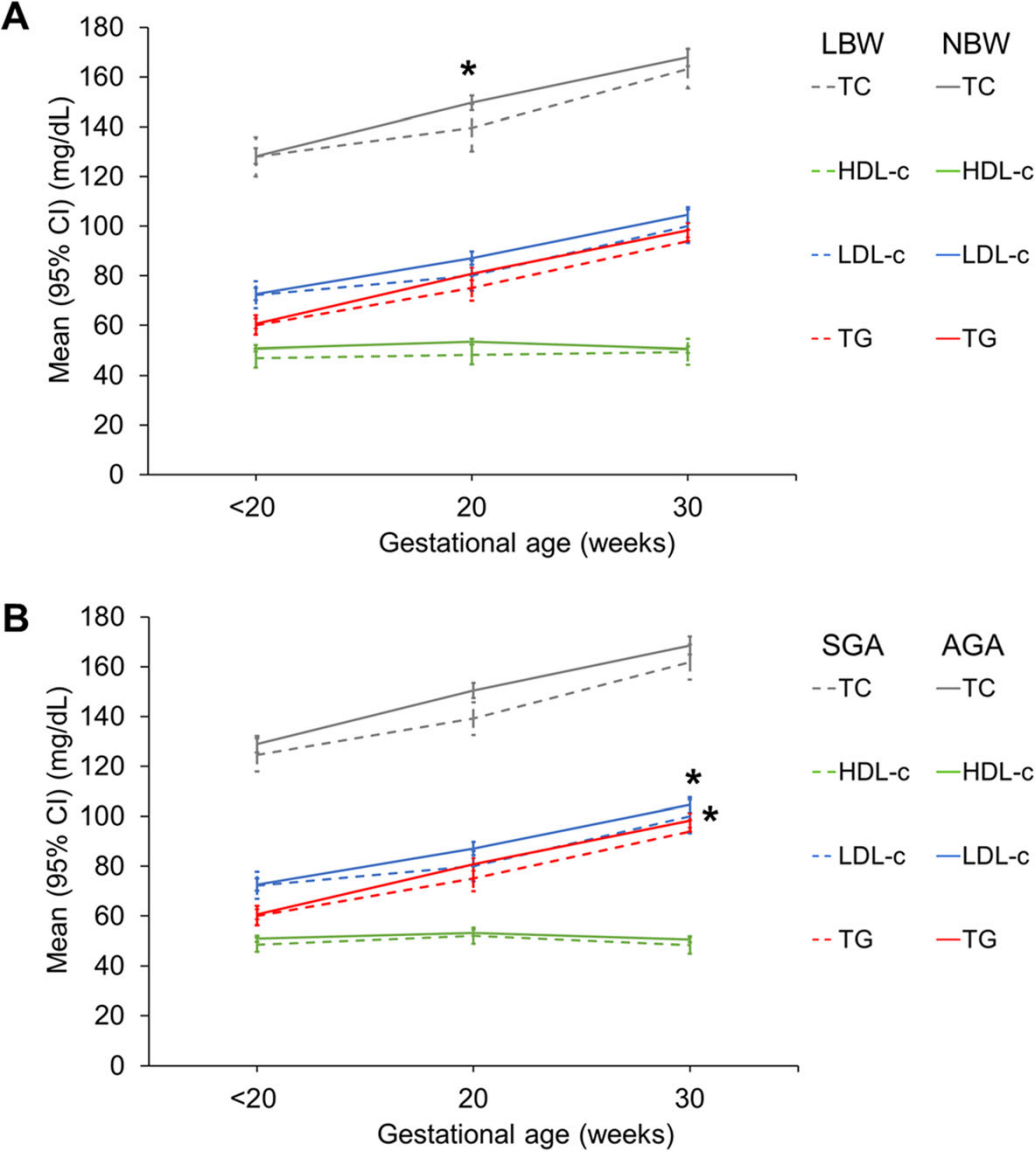
Mean maternal plasma lipids at enrolment, 20 and 30 weeks gestation by LBW and SGA status. Mean maternal lipids were compared by Student’s t-test with Welch correction for unequal sample size between low birth weight (LBW) (< 2.5 kg) and normal birth weight infant (NBW) (≥2.5 kg) and between small-for-gestational-age (SGA) (<10th percentile INTERGROWTH-21ST for birth weight) and adequate-for-gestational-age (AGA) (≥10th percentile INTERGROWTH-21ST for birth weight) infants. Mean total cholesterol (TC) levels were significantly lower at 20 weeks gestation in women with LBW infants compared to women with NBW infants (*P* = 0.048). Women with SGA infants had at 30 weeks gestation lower mean low-density lipoprotein cholesterol (LDL-c) (*P* = 0.033) and triglycerides (TG) (P = 0.048) levels compared to women with AGA infants. Mean (SD) gestational age at enrolment was 13.9 (3.3) weeks gestation

Mean levels of TC, LDL-c and TG increased from enrolment to 30 weeks gestation (all, *P* < 0.001) while mean HDL-c levels increased slightly from enrolment to 20 weeks gestation (51.4 vs 53.1 mg/dL, *P* = 0.003) before decreasing back, by 30 weeks gestation, to similar levels observed at enrolment (53.1 vs 51.2 mg/dL, *P* < 0.001). Longitudinal changes in maternal lipid levels were compared by LBW vs NBW and SGA vs AGA (Fig. 2). Women with LBW infants had lower mean TC levels at 20 weeks gestation compared to those with NBW infants (133.6 vs 149.6 mg/dL, *P* = 0.048) (Fig. 2a). Women with SGA infants had lower mean LDL-c (99.5 vs 105.9 mg/dL, *P* = 0.033), and TG (94.2 vs 99.4 mg/dL, *P* = 0.048) levels at 30 weeks gestation compared to women with AGA infants (Fig. 2b).

Links between maternal lipid levels during pregnancy and the risk of delivering a LBW infant were investigated using regression models (Table 2 and Table 3). In the adjusted analyses, every unit increase in HDL-c at enrolment was associated with a 2.7% (*P* = 0.011) reduction in the relative risk of LBW and every unit increase in TC levels at 20 weeks gestation were associated with a 1.3% reduction in the relative risk of LBW (*P* = 0.002) (Table 2). Women with low (<10th percentile) HDL-c at enrolment or at 20 weeks gestation had a 2.6 (*P* = 0.007) and 3.0 (*P* = 0.003) higher risk of delivering a LBW infant compared to women with referent (10th─90th percentile) HDL-c level, respectively (Table 3).

**Table 2:** Relative risk reduction (RRR) (%) (95% CI) of LBW in association with maternal total, HDL and LDL cholesterol and triglycerides levels at enrolment, 20 and 30 weeks gestation^a^

**Gestation week**	**RRR (%) (95% CI)**	***p-value***	**aRRR (95% CI)**^**b**^	***p-value***
**Total cholesterol**				
Enrolment^c^	0.02 (−0.66, 0.70)	0.944	0.51 (−0.27, 1.3)	0.196
20 weeks	1.1 (0.02, 2.2)	**0.045**	1.3 (0.49, 2.2)	**0.002**
30 weeks	0.31 (− 0.28, 0.89)	0.302	0.30 (− 0.39, 0.98)	0.399
**HDL cholesterol**				
Enrolment^c^	1.9 (−0.10, 3.9)	0.063	2.7 (0.64, 4.8)	**0.011**
20 weeks	2.4 (−0.57, 5.3)	0.112	2.7 (−0.59, 6.0)	0.106
30 weeks	1.1 (−0.95, 3.0)	0.300	0.27 (−1.5, 2.0)	0.763
**LDL cholesterol**				
Enrolment^c^	−0.23 (−1.1, 0.59)	0.581	−0.01 (− 0.96, 0.92)	0.977
20 weeks	0.66 (− 0.33, 1.6)	0.190	1.0 (− 0.04, 2.1)	0.060
30 weeks	0.23 (−0.41, 0.86)	0.486	0.33 (−0.39, 1.1)	0.370
**Triglycerides**				
Enrolment^c^	−0.52 (−1.5, 0.49)	0.316	0.02 (−1.2, 1.2)	0.977
20 weeks	−0.19 (−1.6, 1.2)	0.792	−0.45 (−2.0, 1.1)	0.560
30 weeks	0.41 (−0.40, 1.2)	0.320	0.33 (−0.50, 1.2)	0.436

^a^Relative risk reduction was calculated as % = (1 - RR) × 100. The RR values are presented in additional Table A2

^b^Binary regression models were adjusted with enrolment maternal age, parity, gestational age, hemoglobin concentration, BMI, supplement group, and compliance to supplement during pregnancy, and infant birth sex and birth season

^c^Mean (SD) gestational age at enrolment was 13.9 (3.3) weeks gestation

*Abbreviations*: *HDL* high-density lipoprotein, *LDL* low-density lipoprotein, *(a)RRR* adjusted relative risk reduction

**Table 3:** Relative risk (RR) (95% CI) of LBW by maternal total, HDL and LDL cholesterol and triglycerides levels divided in percentile groups, at enrolment, 20 and 30 weeks gestation^a^

**Gestation week**	**mg/dL**	**LBW, n (%)**	**Unadjusted**		**Adjusted**^**b**^	
			**RR (95%CI)**	***p-value***	**aRR (95%CI)**	***p-value***
**Total cholesterol**						
**Enrolment**^**c**^						
Low (<10th)	< 88.6	2 (4.1)	0.42 (0.10, 1.7)	0.221	0.54 (0.14, 2.2)	0.384
Ref (10th─90th)	88.6─170.5	38 (9.7)	Ref		Ref	
High (>90th)	> 170.5	1 (2.0)	0.21 (0.03, 1.5)	0.119	0.17 (0.02, 1.2)	0.070
**20 weeks**						
Low (<10th)	< 112.9	3 (7.1)	0.90 (0.29, 2.9)	0.865	1.2 (0.78, 1.8)	0.451
Ref (10th─90th)	112.9─185.6	27 (7.9)	Ref		Ref	
High (>90th)	> 185.6	0 (0.0)	–	–	–	–
**30 weeks**						
Low (<10th)	< 123.6	2 (4.1)	0.41 (0.10, 1.6)	0.195	0.38 (0.09, 1.6)	0.197
Ref (10th─90th)	123.6─214.2	40 (10.2)	Ref		Ref	
High (>90th)	> 214.2	1 (2.0)	0.20 (0.03, 1.4)	0.103	0.20 (0.03, 1.6)	0.139
**HDL cholesterol**						
**Enrolment**^**c**^						
Low (<10th)	< 33.6	7 (13)	1.6 (0.74, 3.4)	0.229	2.6 (1.3, 5.3)	**0.007**
Ref (10th─90th)	33.6─68.4	34 (8.1)	Ref		Ref	
High (>90th)	> 68.4	2 (3.8)	0.47 (0.11, 1.9)	0.283	0.50 (0.12, 2.1)	0.337
**20 weeks**						
Low (<10th)	< 36.3	6 (12.8)	2.0 (0.85, 4.6)	0.115	3.0 (1.5, 6.4)	**0.003**
Ref (10th─90th)	36.3─70	24 (6.5)	Ref		Ref	
High (>90th)	> 70	1 (2.1)	0.30 (0.05, 2.4)	0.270	0.40 (0.07, 2.6)	0.341
**30 weeks**						
Low (<10th)	< 33.3	6 (11.5)	1.3 (0.58, 3.0)	0.507	0.90 (0.37, 2.2)	0.813
Ref (10th─90th)	33.3─ 68.4	35 (8.8)	Ref		Ref	
High (>90th)	> 68.4	4 (7.3)	0.80 (0.31, 2.3)	0.716	0.90 (0.41, 1.9)	0.753
**LDL cholesterol**						
**Enrolment**^**c**^						
Low (<10th)	< 43.3	3 (5.1)	0.58 (0.19, 1.8)	0.357	0.63 (0.21, 1.8)	0.390
Ref (10th─90th)	43.3─106	36 (8.7)	Ref		Ref	
High (>90th)	> 106	4 (7.4)	0.85 (0.31, 2.3)	0.748	0.68 (0.24, 1.9)	0.467
**20 weeks**						
Low (<10th)	< 53.4	1 (2.2)	0.28 (0.04, 2.0)	0.203	0.44 (0.10, 2.0)	0.280
Ref (10th─90th)	53.4─122.6	29 (7.8)	Ref		Ref	
High (>90th)	> 122.6	1 (2.1)	0.30 (0.04, 2.0)	0.196	0.30 (0.04, 2.2)	0.229
**30 weeks**						
Low (<10th)	< 62.3	2 (3.9)	0.37 (0.09, 1.5)	0.159	0.42 (0.10, 1.7)	0.229
Ref (10th─90th)	62.3─146.6	43 (10.6)	Ref		Ref	
High (>90th)	> 146.6	0 (0.0)	–	–	–	–
**Triglycerides**						
**Enrolment**^**c**^						
Low (<10th)	< 35.4	1 (2)	0.23 (0.03, 1.7)	0.147	0.26 (0.03, 2.0)	0.193
Ref (10th─90th)	35.4─88.6	37 (8.7)	Ref		Ref	
High (>90th)	> 88.6	5 (9.3)	1.1 (0.44, 2.6)	0.896	0.97 (0.42, 2.2)	0.937
**20 weeks**						
Low (<10th)	< 53.1	3 (5.3)	0.70 (0.22, 2.2)	0.553	0.94 (0.28, 3.2)	0.926
Ref (10th─90th)	53.1─112.5	27 (7.5)	Ref		Ref	
High (>90th)	> 112.5	2 (4.3)	0.60 (0.10, 2.3)	0.432	0.60 (0.20, 2.7)	0.539
**30 weeks**						
Low (<10th)	< 65.5	2 (4.2)	0.41 (0.10, 1.6)	0.203	0.44 (0.11, 1.7)	0.233
Ref (10th─90th)	65.5─134.6	42 (10.3)	Ref		Ref	
High (>90th)	> 134.6	1 (2.0)	0.20 (0.03, 1.4)	0.098	0.20 (0.02, 1.5)	0.116

^a^Binary regression models between the risk of LBW and maternal lipid levels divided by percentile groups; <10th percentile (low), 10th─90th (reference, Ref), >90th percentile (high)

^b^Adjusted relative risk (aRR) with enrolment maternal age, parity, gestational age, hemoglobin concentration, BMI, supplement group, and compliance to supplement during pregnancy, and infant birth sex and birth season

^c^Mean (SD) gestational age at enrolment was 13.9 (3.3) weeks gestation

*Abbreviations*: *HDL* high-density lipoprotein, *LDL* low-density lipoprotein, *LBW* low birth weight, *(a)RR* adjusted relative risk

Tables 4 and 5 present associations between maternal lipid levels and risk of SGA. In the unadjusted analyses, an association between increased LDL-c levels at 30 weeks gestation and a reduced risk of SGA was detected (RRR = 0.38%, *P* = 0.048) (Table 4) but no other associations were observed. In both the unadjusted and adjusted analyses, women with high (>90th percentile) LDL-c at 30 weeks gestation had a 55% (*P* = 0.017) lower risk of delivering an SGA infant compared to women with referent (10th─90th percentile) LDL-c levels (Table 5). In the unadjusted analyses, women with high (>90th percentile) TG at 30 weeks gestation had a 48% (*P* = 0.035) lower risk of delivering an SGA infant compared to women with referent (10th─90th percentile) TG, however, this association was lost following adjustment for confounding factors (Table 5).

**Table 4:** Relative risk reduction (RRR) (%) (95%CI) of SGA in association with maternal total, HDL and LDL cholesterol and triglycerides levels at enrolment, 20 and 30 weeks gestation^a^

**Gestation week**	**RRR (%) (95% CI)**	***p-value***	**aRRR (95% CI)**^**b**^	***p-value***
**Total cholesterol**				
Enrolment^c^	0.13 (−0.24, 0.51)	0.489	0.11 (−0.28, 0.5)	0.576
20 weeks	0.18 (−0.30, 0.65)	0.463	−0.01 (− 0.49, 0.47)	0.975
30 weeks	0.29 (− 0.06, 0.63)	0.101	0.16 (− 0.21, 0.52)	0.395
**HDL cholesterol**				
Enrolment^c^	0.05 (−0.83, 0.92)	0.919	0.32 (−0.63, 1.3)	0.507
20 weeks	−0.04 (−1.2, 1.1)	0.947	0.15 (−1.1, 1.3)	0.808
30 weeks	0.02 (−0.93, 0.96)	0.968	−0.08 (−1.01, 0.85)	0.873
**LDL cholesterol**				
Enrolment^c^	0.12 (−0.33, 0.57)	0.604	0.00 (−0.45, 0.45)	0.991
20 weeks	0.09 (−0.42, 0.61)	0.723	−0.07 (− 0.60, 0.45)	0.785
30 weeks	0.38 (0.00, 0.76)	**0.048**	0.26 (−0.13, 0.65)	0.193
**Triglycerides**				
Enrolment^c^	0.13 (−0.43, 0.69)	0.640	0.11 (−0.49, 0.69)	0.725
20 weeks	0.33 (−0.25, 0.91)	0.261	0.19 (−0.38, 0.75)	0.514
30 weeks	0.42 (−0.03, 0.87)	0.068	0.32 (−0.14, 0.77)	0.168

^a^Relative risk reduction was calculated as % = (1 - RR) × 100. The RR values are presented in additional Table A3

^b^Binary regression models were adjusted with enrolment maternal age, parity, hemoglobin concentration, BMI, supplement group, and compliance to supplement during pregnancy, and infant birth sex and birth season

^c^Mean (SD) gestational age at enrolment was 13.9 (3.3) weeks gestation

*Abbreviations*: *HDL* high-density lipoprotein, *LDL* low-density lipoprotein, *(a)RRR* adjusted relative risk reduction, *SGA* small-for-gestational-age

**Table 5:** Relative risk (RR) (95% CI) of SGA by maternal total, HDL and LDL cholesterol and triglycerides levels divided in percentile groups, at enrolment, 20 and 30 weeks gestation^a^

**Gestation week**	**mg/dL**	**SGA,*n*(%**)	**Unadjusted**		**Adjusted**^**b**^	
			**RR (95%CI)**	***p-value***	**aRR (95%CI)**	***p-value***
**Total cholesterol**						
**Enrolment**^**c**^						
Low (<10th)	< 88.6	13 (26.5)	0.78 (0.48, 1.3)	0.312	0.82 (0.51, 1.3)	0.422
Ref (10th─90th)	88.6─170.5	133 (34.1)	Ref		Ref	
High (>90th)	> 170.5	11 (22.5)	0.66 (0.38, 1.1)	0.128	0.73 (0.42, 1.2)	0.246
**20 weeks**						
Low (<10th)	< 112.9	16 (38.1)	1.2 (0.79, 1.8)	0.401	1.1 (0.69, 1.8)	0.672
Ref (10th─90th)	112.9─185.6	109 (31.9)	Ref		Ref	
High (>90th)	> 185.6	11 (25.6)	0.80 (0.47, 1.4)	0.419	0.86 (0.51, 1.5)	0.576
**30 weeks**						
Low (<10th)	< 123.6	15 (30.6)	0.93 (0.59, 1.4)	0.742	0.82 (0.51, 1.3)	0.427
Ref (10th─90th)	123.6─214.2	129 (33)	Ref		Ref	
High (>90th)	> 214.2	10 (20)	0.61 (0.34, 1.1)	0.087	0.65 (0.36, 1.2)	0.142
**HDL cholesterol**						
**Enrolment**^**c**^						
Low (<10th)	< 33.6	18 (33.3)	1.0 (0.67, 1.5)	0.981	1.2 (0.82, 1.7)	0.352
Ref (10th─90th)	33.6─68.4	139 (33.2)	Ref		Ref	
High (>90th)	> 68.4	15 (28.3)	0.85 (0.54, 1.3)	0.489	0.82 (0.50, 1.4)	0.443
**20 weeks**						
Low (<10th)	< 36.3	19 (40.4)	1.4 (0.95, 2.0)	0.095	1.7 (1.3, 2.3)	0.052
Ref (10th─90th)	36.3─70	108 (29.2)	Ref		Ref	
High (>90th)	> 70	16 (34)	1.2 (0.76, 1.8)	0.482	1.2 (0.81, 1.9)	0.312
**30 weeks**						
Low (<10th)	< 33.3	15 (28.9)	0.88 (0.56, 1.4)	0.580	0.80 (0.51, 1.3)	0.337
Ref (10th─90th)	33.3─ 68.4	131 (32.8)	Ref		Ref	
High (>90th)	> 68.4	17 (30.9)	0.94 (0.62, 1.4)	0.787	0.88 (0.57, 1.3)	0.543
**LDL cholesterol**						
**Enrolment**^**c**^						
Low (<10th)	< 43.3	18 (30.5)	0.92 (0.61, 1.4)	0.688	0.89 (0.59, 1.4)	0.601
Ref (10th─90th)	43.3─106	137 (33.2)	Ref		Ref	
High (>90th)	> 106	17 (31.5)	0.95 (0.63, 1.4)	0.806	1.0 (0.66, 1.5)	0.991
**20 weeks**						
Low (<10th)	< 53.4	15 (32.6)	1.1 (0.69, 1.7)	0.733	1.05 (0.65, 1.7)	0.851
Ref (10th─90th)	53.4─122.6	112 (30.2)	Ref		Ref	
High (>90th)	> 122.6	16 (34)	1.1 (0.74, 1.7)	0.582	1.3 (0.84, 1.9)	0.265
**30 weeks**						
Low (<10th)	< 62.3	18 (35.3)	1.0 (0.70, 1.5)	0.843	0.86 (0.54, 1.4)	0.543
Ref (10th─90th)	62.3─146.6	137 (33.9)	Ref		Ref	
High (>90th)	> 146.6	8 (15.4)	0.45 (0.24, 0.90)	**0.018**	0.45 (0.23, 0.90)	**0.017**
**Triglycerides**						
**Enrolment**^**c**^						
Low (<10th)	< 35.4	12 (24.5)	0.73 (0.44, 1.2)	0.229	0.70 (0.42, 1.2)	0.181
Ref (10th─90th)	35.4─88.6	142 (33.5)	Ref		Ref	
High (>90th)	> 88.6	18 (33.3)	1.0 (0.67, 1.5)	0.982	0.95 (0.62, 1.5)	0.798
**20 weeks**						
Low (<10th)	< 53.1	20 (35.1)	1.1 (0.74, 1.6)	0.653	1.1 (0.75, 1.6)	0.649
Ref (10th─90th)	53.1─112.5	116 (32.1)	Ref		Ref	
High (>90th)	> 112.5	9 (19.2)	0.60 (0.30, 1.1)	0.095	0.70 (0.40, 1.2)	0.195
**30 weeks**						
Low (<10th)	< 65.5	15 (31.3)	0.92 (0.59, 1.4)	0.709	0.83 (0.53, 1.3)	0.444
Ref (10th─90th)	65.5─134.6	139 (34)	Ref		Ref	
High (>90th)	> 134.6	9 (17.7)	0.52 (0.28, 0.99)	**0.035**	0.54 (0.29, 1.0)	0.068

^a^Binary regression models between the risk of SGA and maternal lipid levels divided by percentile groups; <10th percentile (low), 10th─90th (reference, Ref), >90th percentile (high)

^b^Adjusted relative risk were adjusted with enrolment maternal age, parity, hemoglobin concentration, BMI, supplement group, and compliance to supplement during pregnancy, and infant birth sex and birth season

^c^Mean (SD) gestational age at enrolment was 13.9 (3.3) weeks gestation

*Abbreviations*: *HDL* high-density lipoprotein, *LDL* low-density lipoprotein, *(a)RRR* adjusted relative risk reduction, *SGA* small-for-gestational-age

Linear regression models were used to examine associations between maternal lipid levels during pregnancy and birth weight (Table 6 and Table 7). In the unadjusted analyses, increased levels of TC and LDL-c at 20 and 30 weeks gestation and of TG at 30 weeks gestation were all associated with a higher infant birth weight (all, *P* < 0.05) (Table 6). After adjustment with confounding factors, only increased levels of TC at 20 weeks gestation (β = 1.3, *P* = 0.027) and of TC (β = 1.2, *P* = 0.006) and LDLc (β = 1.5, *P* = 0.002) at 30 weeks gestation remained associated with a higher birth weight (Table 6). In both the unadjusted and adjusted analyses, women with high (>90th percentile) levels of TC, LDL-c or TG at 30 weeks gestation had infants with higher birth weights compared with women with referent (10th─90th percentile) levels (*P* < 0.05) (Table 7).

**Table 6:** Beta coefficients (95% CI) for maternal total, HDL and LDL cholesterol and triglycerides levels, at enrolment, 20 and 30 weeks gestation, in association with birth weight in grams

**Gestation week**	**Unadjusted**			**Adjusted**^**a**^		
	**R**^**2**^	**β (95% CI)**	***p-value***	**R**^**2**^	**β (95% CI)**	***p-value***
**Total cholesterol**						
Enrolment^b^	0.012	0.90 (−0.01, 1.9)	0.052	0.152	1.1 (− 0.01, 2.2)	0.053
20 weeks	0.010	1.3 (0.20, 2.4)	**0.026**	0.130	1.3 (0.20, 2.5)	**0.027**
30 weeks	0.012	1.1 (0.30, 1.9)	**0.006**	0.098	1.2 (0.30, 2.0)	**0.006**
**HDL cholesterol**						
Enrolment^b^	0.002	0.10 (−2.2, 2.4)	0.933	0.140	0.66 (−2.0, 3.3)	0.621
20 weeks	0.001	1.0 (−1.5, 3.6)	0.427	0.113	1.5 (−1.3, 4.2)	0.295
30 weeks	0.001	0.80 (− 1.5, 3.0)	0.500	0.086	0.90 (−1.5, 3.3)	0.457
**LDL cholesterol**						
Enrolment^b^	0.009	1.0 (−0.10, 2.2)	0.080	0.142	0.80 (− 0.40, 2.1)	0.206
20 weeks	0.009	1.3 (0.10, 2.5)	**0.033**	0.119	1.1 (−0.10, 2.4)	0.082
30 weeks	0.018	1.5 (0.60, 2.4)	**0.001**	0.104	1.5 (0.50, 2.4)	**0.002**
**Triglycerides**						
Enrolment^b^	0.003	0.40 (−1.1, 2.0)	0.589	0.139	0.80 (− 1.0, 2.7)	0.385
20 weeks	0.002	0.70 (−0.60, 2.1)	0.292	0.119	0.50 (−1.0, 2.0)	0.511
30 weeks	0.007	1.1 (0.10, 2.1)	**0.039**	0.093	1.0 (−0.10, 2.1)	0.068

^a^Linear regression models were adjusted with maternal age, parity, gestational age, hemoglobin concentration, BMI, supplement group, and compliance to supplement during pregnancy, and infant birth sex and birth season

^b^Mean (SD) gestational age at enrolment was 13.9 (3.3) weeks gestation

*Abbreviations*: *HDL* high-density lipoprotein, *LDL* low-density lipoprotein

**Table 7:** Mean differences in birth weight by percentile groups of maternal total, HDL and LDL cholesterol and triglycerides levels*,* at enrolment, 20 and 30 weeks gestation^a^

Gestation week	mg/dL	***N***	**Unadjusted**		**Adjusted**^**b**^	
			Mean difference (95% CI) in birth weight (g)	***p-value***	Mean difference (95% CI) in birth weight (g)	***p-value***
**Total cholesterol**						
**Enrolment**^**c**^						
Low (<10th)	< 88.6	49	3.2 (− 108.4, 114.8)	0.955	4.1 (− 109.3, 117.4)	0.944
Ref (10th─90th)	88.6─170.5	390	Ref		Ref	
High (>90th)	> 170.5	49	62.6 (−45.7, 170.9)	0.257	33.0 (−80.8, 146.9)	0.569
**20 weeks**						
Low (<10th)	< 112.9	42	−21.0 (− 131.2, 89.2)	0.708	−31.3 (− 142.3, 79.7)	0.580
Ref (10th─90th)	112.9─185.6	342	Ref		Ref	
High (>90th)	> 185.6	43	109.8 (−1.2, 220.8)	0.052	110.1 (−9.1, 229.3)	0.070
**30 weeks**						
Low (<10th)	< 123.6	49	−51.2 (−130.1, 27.7)	0.203	−45.3 (− 126.0, 35.4)	0.270
Ref (10th─90th)	123.6─214.2	391	Ref		Ref	
High (>90th)	> 214.2	50	108.9 (11.7, 206.1)	**0.028**	111.2 (9.0, 213.3)	**0.033**
**HDL cholesterol**						
**Enrolment**^**c**^						
Low (<10th)	< 33.6	54	−11.9 (−130.9, 107.2)	0.845	−42.1 (− 164.2, 79.9)	0.498
Ref (10th─90th)	33.6─68.4	419	Ref		Ref	
High (>90th)	> 68.4	53	37.1 (−66.9, 141.2)	0.484	37.8 (−73.4, 149.1)	0.504
**20 weeks**						
Low (<10th)	< 36.3	47	−55.0 (− 171.5, 61.5)	0.354	−100.5 (−214.3, 13.4)	0.084
Ref (10th─90th)	36.3─70	370	Ref		Ref	
High (>90th)	> 70	47	−2.5 (− 100.4, 95.5)	0.961	−6.4 (−116.8, 104.0)	0.909
**30 weeks**						
Low (<10th)	< 33.3	52	−31.5 (−133.5, 70.5)	0.544	−14.8 (− 121.2, 91.5)	0.784
Ref (10th─90th)	33.3─ 68.4	400	Ref		Ref	
High (>90th)	> 68.4	55	−9.0 (− 118.2, 100.1)	0.871	5.7 (−103.1, 114.5)	0.918
**LDL cholesterol**						
**Enrolment**^**c**^						
Low (<10th)	< 43.3	59	2.7 (−101.3, 106.7)	0.959	5.2 (−95.1, 105.4)	0.919
Ref (10th─90th)	43.3─106	413	Ref		Ref	
High (>90th)	> 106	54	−6.6 (−114.6, 101.5)	0.905	−27.7 (−139.1, 83.7)	0.626
**20 weeks**						
Low (<10th)	< 53.4	46	−15.4 (− 123.8, 93.0)	0.780	−9.3 (− 119.9, 101.3)	0.868
Ref (10th─90th)	53.4─122.6	371	Ref		Ref	
High (>90th)	> 122.6	47	42.1 (−67.3, 151.4)	0.450	24.2 (−93.8, 142.3)	0.687
**30 weeks**			Ref			
Low (<10th)	< 62.3	51	−51.9 (−141.1, 37.3)	0.254	−54.3 (− 153.1, 44.6)	0.281
Ref (10th─90th)	62.3─146.6	404	Ref		Ref	
High (>90th)	> 146.6	52	122.1 (30.8, 213.4)	**0.009**	125.3 (29.7, 220.8)	**0.010**
**Triglycerides**						
**Enrolment**^**c**^						
Low (<10th)	< 35.4	49	87.4 (−27.6, 202.5)	0.136	92.6 (−22.3, 207.5)	0.114
Ref (10th─90th)	35.4─88.6	424	Ref		Ref	
High (>90th)	> 88.6	54	41.7 (−77.8, 161.2)	0.493	34.9 (−96.6, 166.3)	0.602
**20 weeks**			Ref			
Low (<10th)	< 53.1	57	28.5 (−72.3, 129.3)	0.578	26.5 (−79.6, 132.5)	0.624
Ref (10th─90th)	53.1─112.5	361	Ref		Ref	
High (>90th)	> 112.5	47	89.6 (−21.5, 200.6)	0.114	59.6 (−54.3, 173.5)	0.304
**30 weeks**						
Low (<10th)	< 65.5	48	28.5 (−70.3, 127.3)	0.571	21.0 (−89.5, 131.5)	0.709
Ref (10th─90th)	65.5─134.6	409	Ref		Ref	
High (>90th)	> 134.6	51	126.5 (25.0, 227.9)	**0.015**	121.8 (5.2, 238.4)	**0.041**

^a^Maternal lipid levels were divided by percentile groups; <10th percentile (low), 10th─90th (reference, Ref), >90th percentile (high)

^b^Adjusted mean birth weight with maternal age, parity, gestational age, hemoglobin concentration, BMI, supplement group, and compliance to supplement during pregnancy, and infant birth sex and birth season

^c^Mean (SD) gestational age at enrolment was 13.9 (3.3) weeks gestation

*Abbreviations*: *HDL* high-density lipoprotein, *LDL* low-density lipoprotein

In a sub-analysis, we explored potential interactions between maternal nutritional supplement groups, BMI, lipid levels and infant birth weight outcomes (Additional Tables A4 to A8). Associations were observed between maternal nutritional supplement groups and maternal lipid levels during pregnancy (Table A4). Compared to FeFol (referent/control group), supplementation with PE + MMN was associated with lower HDLc levels at 20 (β = − 3.7, *P* = 0.028) and 30 (β = − 4.3, *P* = 0.013) weeks gestation, and supplementation with PE was associated with higher LDL-c levels (β = 9.3, *P* = 0.034) at 30 weeks gestation (Table A4). We also detected associations between maternal lipid levels and BMI during pregnancy (Table A5). At enrolment, a lower BMI was associated with increased HDL-c levels (β = − 0.025, *P* = 0.021) whereas a higher BMI was associated with increased LDL-c levels (β = 0.012, *P* = 0.034). At 20 and 30 weeks gestation, higher BMIs were linked to increased TG levels (β = 0.011, *P* = 0.018 and β = 0.009, *P* = 0.048, respectively) (Table A5). However, there were no associations between maternal nutritional supplement groups and maternal BMI at any timepoint across pregnancy (Table A6). Additionally, maternal supplement groups were not associated with the risks of LBW or SGA (Table A7) or birth weight (Table A8).

## Discussion

In this cohort of pregnant women from rural Gambia, plasma lipid levels were lower compared to those reported in studies from high-income countries [26–29] or urban areas in LMICs [30], likely reflecting the typical diet in rural Gambia which is low in animal-source foods and consists mainly of carbohydrate-rich staple foods. Low HDL-c levels in the first half of pregnancy were associated with three times the risk of LBW. Increased TC levels from mid-pregnancy were associated with higher birth weight and high LDL-c at 30 weeks gestation was associated with half the risk of SGA. These findings suggest that dietary interventions to improve diet and increase cholesterol levels during pregnancy may promote fetal growth and reduce adverse birth weight-related outcomes.

Consistent with previous studies, we observed an increase in plasma TC, LDL-c and TG levels across pregnancy [10, 16, 17, 26, 27], which is known to be part of a normal physiological response to gestation [28]. However, unlike in studies from higher-income countries [10, 27, 28], our data show a decrease in HDL-c levels from 20 to 30 weeks gestation, following an initial rise across the first half of pregnancy. Similarly, a study conducted in rural India (*n =* 631) reported a decrease in maternal HDL-c levels between 18 and 28 weeks gestation [16]. These observations indicate that dietary intake may influence changes in HDL-c level across pregnancy and specifically the ability to maintain HDL-c levels in the latter part of gestation.

In line with previous research from a range of settings, relationships between maternal TC, HDL-c, LDL-c, and TG levels during pregnancy and size at birth were observed in our study. An association between increased TC levels at 20 weeks gestation and a reduced risk of LBW was detected and increased TC levels at both 20 and 30 weeks’ gestation were associated with higher birth weight. In a study conducted in Nigeria (*n =* 261), women with low TC in early pregnancy had a 2-fold increased risk of delivering an LBW infant compared to women with TC levels within the mid-range [15]. Likewise, in a study conducted in rural India, TC levels at both 18 and 28 weeks gestation were associated with higher infant birth weight [16]. These findings are consistent with the high demand for cholesterol during pregnancy to meet both maternal and fetal needs [10]. Although the primary source of cholesterol for the fetus may be derived from fetal biosynthesis, recent studies demonstrated that placental trophoblast and endothelial cells can effectively transfer maternal cholesterol to the fetus throughout pregnancy, thus impacting on fetal growth and infant birth weight [29–31].

Similar to our findings of an association between low HDL-c in the second trimester and greater risk of LBW, a study conducted in the US (*n =* 1207) reported that low HDL-c measured at 16–27 weeks gestation was associated with a lower birth weight [12]. Low HDL-c levels in non-pregnant populations have been associated with atherogenic lipid profiles and higher risks of cardiovascular disease and mortality [32–34]. During pregnancy, low HDL-c levels have been linked to greater risks of adverse pregnancy outcomes [35], including preterm birth [36], a shorter pregnancy length [27] and pre-eclampsia [35, 37]. One of the main functions of HDL is to promote reverse cholesterol transport from peripheral tissues to the liver for excretion in bile acids. Since HDL presents anti-atherogenic and anti-inflammatory properties, a low level of HDL-c during pregnancy may increase the risk of endothelial damage in uteroplacental and fetal tissues caused by inflammation resulting in placental dysfunction, inadequate supply of oxygen and nutrients to the fetus and intrauterine growth restriction (IUGR) [38, 39].

Additionally, at 30 weeks gestation, we detected associations between high LDL-c and reduced risk of SGA and between increased LDL-c levels and higher infant birth weight. This corroborates the findings of a study conducted in Scotland (*n =* 66) which reported an association between low LDL-c in the first trimester and IUGR [40]. Studies in both animal models and human subjects suggest that fetal growth is supported by the uptake of maternal cholesterol from the circulation by the placenta via various receptors including VLDL and LDL receptors [31, 41, 42]. Therefore, increased maternal LDL-c levels during pregnancy may promote fetal growth and higher infant birth weight.

In accord with previous research, our data show that at 30 weeks gestation women with high TG delivered infants who were on average 122 g heavier than those born to women with referent TG values. And, women with SGA infants had lower mean TG levels compared to those with AGA infants. Notably, Kulkarni et al. reported that a one SD higher TG level at 28 weeks gestation was associated with a 36 g higher birth weight [16] and Jin et al. reported that increased TG levels in the third trimester were associated with a reduced risk of SGA [10]. However, contrary to studies conducted in populations with greater rates of overweight and obesity [12, 17, 19, 43], our data suggests a limited impact of TG on birth weight-related outcomes, which may be linked to the lower levels of TG detected in this cohort and may reflect poor dietary intake of TG [36, 44–46]. Maternal TG and their derived non-esterified fatty acids (NEFA) are mainly obtained through maternal diet and their levels during pregnancy have been shown to directly correlate with fetal lipid levels and fetal growth, thereby impacting birth weight [47].

Although all LBW infants were also born SGA in our study, the results show differences in the relationships between maternal lipids during pregnancy and the risks of LBW and SGA. While increased HDL-c or TC levels in the first half of pregnancy were linked to a reduced risk of LBW, high LDL-c or TG at 30 weeks gestation were related to a reduced risk of SGA. This suggests that the pathophysiological mechanisms linking gestational lipid levels to the risk of LBW may arise early in gestation, during the anabolic phase of pregnancy characterized by an accumulation of fat in maternal adipose tissue [48]. Inadequate levels of anti-atherogenic and anti-inflammatory molecules (i.e. HDL) during this phase may cause lipid obstruction of vascular tissues and inflammation resulting in a cascade of events leading to LBW. Maternal inflammation during pregnancy has been associated with IUGR in a study conducted in rural Nepal (*n* = 653) [49] and with lower birth weight in a cohort (*n* = 144) from Tanzania [50]. In contrast, the pathophysiological mechanisms associated with the risk of SGA may appear later in pregnancy; during the catabolic phase where insulin resistance and placental hormones induce the release of free fatty acids from maternal adipose tissue to meet the heightened fetal energy demands. Low pregnancy weight gain may cause insufficient storage of energy in adipose tissue and supply of cholesterol or TG to the fetus during the catabolic phase of pregnancy. This may reduce fetal growth and result in an infant born SGA. Inadequate weight gain during the third trimester has been linked to IUGR and SGA in a study conducted in rural Bangladesh (*n* = 1463) [51]. Altered adipose tissue and reduced body fat mass have been reported in infants born SGA compared to those born AGA or LGA [52, 53].

Maternal undernutrition during pregnancy causes metabolic stresses which may adversely impact on fetal growth and birth outcomes. In this study and others, underweight women were found more likely to have an LBW or SGA infant compared to women with a normal weight [48–51]. Furthermore, our results show that although maternal nutritional supplementation with a lipid-based supplement (PE or PE + MMN) during pregnancy was associated with altered lipid levels, no direct associations were observed between maternal supplement groups and birth weight outcomes. This may indicate a limited effect of supplementation during pregnancy and a greater role of maternal weight on infant birth weight outcomes in this cohort [54].

The major strengths of this study include the use of a large cohort from a population in rural sub-Saharan Africa, bringing new perspectives into the relationships between maternal lipids and infant birth outcomes in populations where the burden of LBW and SGA is the highest and where research findings are limited. The measurements of maternal plasma TC, HDL-c, LDL-c, and TG levels at three-time points across pregnancy allow a greater understanding of the adaptive process through which maternal lipids impact on fetal growth. The assessment of potential associations using maternal lipid levels as continuous variables and percentile groups with or without adjustment with confounding factors allowed a robust and thorough analysis of the links between gestational lipids and birth weight-related outcomes.

Limitations of this study should be noted, and caution should be taken in the interpretation of the results. This study is a secondary analysis of a clinical trial of nutritional supplementation which by its observational design precludes any conclusion on causal links between maternal lipid levels and infant’s size at birth. Pregnant women in our study were healthy, had a singleton infant and received supplementation and comprehensive antenatal care throughout pregnancy, and therefore may not be representative of all eligible Gambian women. A further limitation of the trial design was the lack of data or samples from late pregnancy to allow lipid measurements after 30 weeks gestation. Such data may have contributed to the understanding of the relationships between gestational lipids and birth outcomes.

## Conclusions

In conclusion, our data from rural sub-Saharan Africa indicate that maternal plasma lipid levels during pregnancy may influence fetal growth, thereby impacting on birth weight and the risks of LBW and SGA. Importantly, our findings suggest that distinct pathophysiological pathways may be involved in the relationships between maternal lipid levels during pregnancy and LBW and SGA outcomes, with differential effects according to the lipid component and the stage of gestation. Additionally, in this cohort of primarily subsistence farming women, underweight women were found at greater risk of having an LBW or SGA infant, highlighting the critical role of maternal nutritional status on birth weight outcomes. Thus, our study supports interventions that monitor and optimize maternal lipid levels across pregnancy while promoting an adequate gestational weight gain.

## Supplementary information

**Additional file 1 Table A1**. Nutritional composition of the allocated daily intake of pregnancy supplements. **Table A2**. Relative risk of LBW (95%CI) associated with maternal total, HDL and LDL cholesterol and triglycerides levels at enrolment, 20 and 30 weeks gestation. **Table A3**. Relative risk of SGA (95%CI) associated with maternal total, HDL and LDL cholesterol and triglycerides levels at enrolment, 20 and 30 weeks’ gestation. **Table A4**. Associations between maternal nutritional supplement groups and maternal total, HDL and LDL cholesterol and triglycerides levels at enrolment, 20 and 30 weeks gestation. **Table A5**. Beta coefficients (95% confidence intervals) of associations between maternal total, HDL, LDL cholesterol and triglycerides levels and BMI at enrolment, 20 and 30 weeks gestation. **Table A6**. Associations between maternal nutritional supplement groups and maternal BMI at enrolment, 20 and 30 weeks gestation. **Table A7**. Relative risk of LBW and SGA by maternal nutritional supplementation groups. **Table A8**. Beta coefficient of the associations between maternal nutritional supplement groups during pregnancy and birth weight.

## Data Availability

The dataset analyzed in this paper is available from the corresponding author on reasonable request, and with appropriate additional ethical approvals, where necessary.

## Abbreviations

AGA: Adequate-for-gestational-age
BMI: Body mass index
CI: Confidence intervals
DSS: Demographic surveillance system
ENID: Early nutrition and immune development
Hb: Hemoglobin
HDL-c: High-density lipoprotein cholesterol
IUGR: Intrauterine growth restriction
LBW: Low birth weight
LDL-c: Low-density lipoprotein cholesterol
LGA: Large-for-gestational-age
LMIC: Low- and middle-income country
MRC: Medical Research Council;
NEFA: Non-esterified fatty acids
SD: Standard deviation
SGA: Small-for-gestational-age
TC: Total cholesterol
TG: Triglycerides
UNICEF: United Nations Children’s Fund
UNU: United Nations University
WHO: World Health Organization
