# Supplementary material for: Maternal plasma lipid levels across pregnancy and the risks of small-for-gestational age and low birth weight: a cohort study from rural Gambia
Source: BMC Pregnancy Childbirth. 2020 Mar 12;20:153. doi: 10.1186/s12884-020-2834-1 (PMC7068879; doi:10.1186/s12884-020-2834-1)
Supplement: Supplementary file 1 — Additional file 1 Table A1. Nutritional composition of the allocated daily intake of pregnancy supplements. Table A2. Relative risk of LBW (95%CI) associated with maternal total, HDL and LDL cholesterol and triglycerides levels at enrolment, 20 and 30 weeks gestation. Table A3. Relative risk of SGA (95%CI) associated with maternal total, HDL and LDL cholesterol and triglycerides levels at enrolment, 20 and 30 weeks’ gestation. Table A4. Associations between maternal nutritional supplement groups and maternal total, HDL and LDL cholesterol and triglycerides levels at enrolment, 20 and 30 weeks gestation. Table A5. Beta coefficients (95% confidence intervals) of associations between maternal total, HDL, LDL cholesterol and triglycerides levels and BMI at enrolment, 20 and 30 weeks gestation. Table A6. Associations between maternal nutritional supplement groups and maternal BMI at enrolment, 20 and 30 weeks gestation. Table A7. Relative risk of LBW and SGA by maternal nutritional supplementation groups. Table A8. Beta coefficient of the associations between maternal nutritional supplement groups during pregnancy and birth weight. [file 12884_2020_2834_MOESM1_ESM.docx]

**Table A1. Nutritional composition of the allocated daily intake of pregnancy supplements**

|  | **Tablets** | | **LNS** | |
| --- | --- | --- | --- | --- |
| **Nutrients** | **FeFol** | **MMN** | **PE** | **MMN + PE** |
| Iron (mg) | 60 | 60 | 60 | 60 |
| Folate (μg) | 400 | 400 | 400 | 400 |
| Vitamin A (RE μg) |  | 1600 | *2.85* | 1600 |
| Vitamin D (IU) |  | 400 | *-* | 400 |
| Vitamin E (mg) |  | 20 | *4.2* | 20 |
| Vitamin C (mg) |  | 140 | *2.25* | 140 |
| Vitamin B1 (mg) |  | 2.8 | *0.3* | 2.8 |
| Vitamin B2 (mg) |  | 2.8 | *0.45* | 2.8 |
| Niacin (mg) |  | 36 | *1.35* | 36 |
| Vitamin B6 (mg) |  | 2.8 | *0.15* | 2.8 |
| Vitamin B12 (μg) |  | 5.2 | *0.1* | 5.2 |
| Zinc (mg) |  | 30 | *3.3* | 30 |
| Copper (mg) |  | 4 | *1.05* | 4 |
| Selenium (μg) |  | 130 | *6.15* | 130 |
| Iodine (μg) |  | 300 | *2.6* | 300 |
| Energy (kcal) |  |  | 746 | 746 |
| Protein (g) |  |  | 20.8 | 20.8 |
| Lipids (g) |  |  | 52.6 | 52.6 |

Abbreviations: FeFol, iron folic acid; LNS, lipid-based nutrient supplement; MMN, multiple micronutrients; PE, protein energy.

Data in italics represent natural micronutrients content in PE which is made from the food base ingredients including soya, maize flour, sorghum, dried skimmed milk power, soybean oil and sugar.

**Table A2.** **Relative risk of LBW (95%CI) associated with maternal total, HDL and LDL cholesterol and triglycerides levels at enrolment, 20 and 30 weeks gestation.**

| **Gestation week** | **RR (95% CI)** | ***p-value*** |  | **aRR (95% CI)^a^** | ***p-value*** |
| --- | --- | --- | --- | --- | --- |
| **Total cholesterol** |  |  |  |  |  |
| Enrolment^b^ | 1.000 (0.993, 1.007) | 0.944 |  | 0.995 (0.987, 1.003) | 0.196 |
| 20 weeks | 0.989 (0.978, 1.000) | **0.045** |  | 0.987 (0.978, 0.995) | **0.002** |
| 30 weeks | 0.997 (0.991, 1.003) | 0.302 |  | 0.997 (0.990, 1.004) | 0.399 |
| HDL cholesterol |  |  |  |  |  |
| Enrolment^b^ | 0.981 (0.961, 1.001) | 0.063 |  | 0.973 (0.952, 0.994) | **0.011** |
| 20 weeks | 0.976 (0.947, 1.006) | 0.112 |  | 0.973 (0.940, 1.006) | 0.106 |
| 30 weeks | 0.989 (0.970, 1.009) | 0.300 |  | 0.997 (0.980, 1.015) | 0.763 |
| LDL cholesterol |  |  |  |  |  |
| Enrolment^b^ | 1.002 (0.994, 1.011) | 0.581 |  | 1.000 (0.991, 1.010) | 0.977 |
| 20 weeks | 0.993 (0.984, 1.003) | 0.190 |  | 0.990 (0.979, 1.000) | 0.060 |
| 30 weeks | 0.998 (0.991, 1.004) | 0.486 |  | 0.997 (0.989, 1.004) | 0.370 |
| Triglycerides |  |  |  |  |  |
| Enrolment^b^ | 1.005 (0.995, 1.015) | 0.316 |  | 1.000 (0.988, 1.012) | 0.977 |
| 20 weeks | 1.002 (0.988, 1.016) | 0.792 |  | 1.005 (0.989, 1.020) | 0.560 |
| 30 weeks | 0.996 (0.988, 1.004) | 0.320 |  | 0.997 (0.988, 1.005) | 0.436 |

^a^Binary regression models were adjusted with enrolment maternal age, parity, gestational age, hemoglobin concentration, BMI, supplement group, and compliance to supplement during pregnancy, and infant birth sex and birth season.

^b^Mean (SD) gestational age at enrolment was 13.9 (3.3) weeks gestation.

Abbreviations: HDL, high-density lipoprotein; LDL, low-density lipoprotein, (a)RR, adjusted relative risk.

**Table A3. Relative risk of SGA (95%CI) associated with maternal total, HDL and LDL cholesterol and triglycerides levels at enrolment, 20 and 30 weeks’ gestation**

| **Gestation week** | **RR (%) (95% CI)** | ***p-value*** |  | **aRR (95% CI)^a^** | ***p-value*** |
| --- | --- | --- | --- | --- | --- |
| **Total cholesterol** |  |  |  |  |  |
| Enrolment^b^ | 0.999 (0.995, 1.002) | 0.489 |  | 0.999 (0.995, 1.003) | 0.576 |
| 20 weeks | 0.998 (0.993, 1.003) | 0.463 |  | 1.000 (0.995, 1.005) | 0.975 |
| 30 weeks | 0.997 (0.994, 1.001) | 0.101 |  | 0.998 (0.995, 1.002) | 0.395 |
| HDL cholesterol |  |  |  |  |  |
| Enrolment^b^ | 1.000 (0.991, 1.008) | 0.919 |  | 0.997 (0.987, 1.006) | 0.507 |
| 20 weeks | 1.000 (0.989, 1.011) | 0.947 |  | 0.999 (0.987, 1.011) | 0.808 |
| 30 weeks | 1.000 (0.990, 1.009) | 0.968 |  | 1.001 (0.992, 1.010) | 0.873 |
| LDL cholesterol |  |  |  |  |  |
| Enrolment^b^ | 0.999 (0.994, 1.003) | 0.604 |  | 1.000 (0.995, 1.004) | 0.991 |
| 20 weeks | 0.999 (0.994, 1.004) | 0.723 |  | 1.001 (0.996, 1.006) | 0.785 |
| 30 weeks | 0.996 (0.992, 1.000) | **0.048** |  | 0.997 (0.994, 1.001) | 0.193 |
| Triglycerides |  |  |  |  |  |
| Enrolment^b^ | 0.999 (0.993, 1.004) | 0.640 |  | 0.999 (0.993, 1.005) | 0.725 |
| 20 weeks | 0.997 (0.991, 1.002) | 0.261 |  | 0.998 (0.993, 1.004) | 0.514 |
| 30 weeks | 0.996 (0.991, 1.000) | 0.068 |  | 0.997 (0.992, 1.001) | 0.168 |

^a^Binary regression models were adjusted with enrolment maternal age, parity, hemoglobin concentration, BMI, supplement group, and compliance to supplement during pregnancy, and infant birth sex and birth season.

^b^Mean (SD) gestational age at enrolment was 13.9 (3.3) weeks gestation.

Abbreviations: HDL, high-density lipoprotein; LDL, low-density lipoprotein, (a)RR, adjusted relative risk.

**Table A4.** **Associations between maternal nutritional supplement groups and maternal total, HDL and LDL cholesterol and triglycerides levels at enrolment, 20 and 30 weeks gestation**

| **Lipids** | **β (95% CI)** | ***p-value*** |  | **β (95% CI)** | ***p-value*** |
| --- | --- | --- | --- | --- | --- |
|  | **Total cholesterol** | |  | **LDL cholesterol** | |
| **Enrolment^a^** |  |  |  |  |  |
| FeFol | Ref |  |  | Ref |  |
| MMN | -2.0 (-10.3, 6.4) | 0.644 |  | -1.2 (-7.7 5.2) | 0.708 |
| PE | 3.5 (-5.1, 12.1) | 0.427 |  | 2.5 (-4.1, 9.0) | 0.465 |
| PE+MMN | -0.78 (-9.2, 7.6) | 0.856 |  | 3.0 (-3.5, 9.5) | 0.363 |
| **20 weeks** |  |  |  |  |  |
| FeFol | Ref |  |  | Ref |  |
| MMN | 2.1 (-5.9, 10.0) | 0.611 |  | 2.5 (-4.4, 9.3) | 0.478 |
| PE | 1.8 (-6.4, 10.1) | 0.663 |  | 2.6 (-4.6, 9.8) | 0.482 |
| PE+MMN | -0.76 (-8.8, 7.2) | 0.853 |  | 4.1 (-2.9, 11.0) | 0.248 |
| **30 weeks** |  |  |  |  |  |
| FeFol | Ref |  |  | Ref |  |
| MMN | 1.4 (-7.6, 10.5) | 0.756 |  | 2.6 (-5.4, 10.6) | 0.528 |
| PE | 6.3 (-3.1, 15.8) | 0.189 |  | 9.0 (0.69, 17.4) | **0.034** |
| PE+MMN | 6.1 (-3.1, 15.4) | 0.193 |  | 6.8 (-1.4, 14.9) | 0.103 |
|  | **HDL cholesterol** | |  | **Triglycerides** | |
| **Enrolment^a^** |  |  |  |  |  |
| FeFol | Ref |  |  | Ref |  |
| MMN | -1.0 (-4.4, 2.4) | 0.548 |  | 3.0 (-2.2, 8.2) | 0.251 |
| PE | 0.64 (-2.8, 4.1) | 0.718 |  | 1.1 (-4.2, 6.4) | 0.686 |
| PE+MMN | -3.3 (-6.7, 0.07) | 0.055 |  | 0.65 (-4.5, 5.8) | 0.805 |
| **20 weeks** |  |  |  |  |  |
| FeFol | Ref |  |  | Ref |  |
| MMN | -1.0 (-4.3, 2.3) | 0.545 |  | 2.4 (-4.0, 8.7) | 0.470 |
| PE | 1.0 (-2.4, 4.4) | 0.565 |  | -1.6 (-8.3, 5.1) | 0.644 |
| PE+MMN | -3.7 (-7.0, -0.39) | **0.028** |  | -0.19 (-6.7, 6.3) | 0.953 |
| **30 weeks** |  |  |  |  |  |
| FeFol | Ref |  |  | Ref |  |
| MMN | -2.9 (-6.2, 0.44) | 0.089 |  | -0.95 (-8.1, 6.2) | 0.794 |
| PE | -1.5 (-5.0, 2.0) | 0.391 |  | 0.07 (-7.4, 7.5) | 0.985 |
| PE+MMN | -4.3 (-7.7, -0.93) | **0.013** |  | -0.09 (-7.4, 7.2) | 0.981 |

^a^Mean (SD) gestational age at enrolment was 13.9 (3.3) weeks gestation.

Abbreviations: FeFol, iron folic acid; HDL, high-density lipoprotein; LDL, low-density lipoprotein, MMN, multiple micronutrients; PE, protein energy.

**Table A5. Beta coefficients (95% confidence intervals) of associations between maternal total, HDL, LDL cholesterol and triglycerides levels and BMI at enrolment, 20 and 30 weeks gestation ^a^**

| **BMI** | **Lipids** | **β (95% CI)** | ***p-value*** |
| --- | --- | --- | --- |
|  | **Total cholesterol** |  |  |
| Enrolment | Enrolment | 0.006 (-0.004, 0.015) | 0.231 |
| 20 weeks | Enrolment | -0.001 (-0.011, 0.009) | 0.793 |
| 30 weeks | Enrolment | 0.002 (-0.006, 0.010) | 0.583 |
| 20 weeks | 20 weeks | 0.003 (-0.007, 0.012) | 0.572 |
| 30 weeks | 20 weeks | 0.006 (-0.001, 0.013) | 0.083 |
| 30 weeks | 30 weeks | 0.006 (-0.001, 0.012) | 0.102 |
|  | **HLD cholesterol** |  |  |
| Enrolment | Enrolment | -0.025 (-0.046, -0.004) | **0.021** |
| 20 weeks | Enrolment | -0.015 (-0.040, 0.009) | 0.223 |
| 30 weeks | Enrolment | -0.001 (-0.021, 0.018) | 0.883 |
| 20 weeks | 20 weeks | -0.011 (-0.035, 0.012) | 0.345 |
| 30 weeks | 20 weeks | 0.002 (-0.016, 0.020) | 0.806 |
| 30 weeks | 30 weeks | 0.006 (-0.012, 0.023) | 0.528 |
|  | **LDL cholesterol** |  |  |
| Enrolment | Enrolment | 0.012 (0.001, 0.023) | **0.034** |
| 20 weeks | Enrolment | -0.001 (-0.012, 0.009) | 0.833 |
| 30 weeks | Enrolment | 0.000 (-0.008, 0.009) | 0.929 |
| 20 weeks | 20 weeks | 0.002 (-0.007, 0.012) | 0.657 |
| 30 weeks | 20 weeks | 0.006 (-0.002, 0.013) | 0.166 |
| 30 weeks | 30 weeks | 0.004 (-0.003, 0.012) | 0.276 |
|  | **Triglycerides** |  |  |
| Enrolment | Enrolment | 0.006 (-0.007, 0.019) | 0.345 |
| 20 weeks | Enrolment | 0.008 (-0.006, 0.022) | 0.265 |
| 30 weeks | Enrolment | 0.008 (-0.002, 0.019) | 0.107 |
| 20 weeks | 20 weeks | 0.009 (-0.004, 0.022) | 0.193 |
| 30 weeks | 20 weeks | 0.011 (0.002, 0.019) | **0.018** |
| 30 weeks | 30 weeks | 0.009 (0.000, 0.017) | **0.048** |

^a^Mean (SD) gestational age at enrolment was 13.9 (3.3) weeks gestation.

Abbreviations: BMI, body mass index, HDL, high-density lipoprotein; LDL, low-density lipoprotein.

**Table A6. Associations between maternal nutritional supplement groups and maternal BMI at enrolment, 20 and 30 weeks gestation**

|  | **BMI** | |
| --- | --- | --- |
| **Supplement group** | **β (95% CI)** | ***p-value*** |
| **Enrolment^a^** |  |  |
| FeFol | Ref |  |
| MMN | 0.09 (-0.69, 0.87) | 0.821 |
| PE | 0.24 (-0.57, 1.0) | 0.567 |
| PE+MMN | 0.57 (-0.26, 1.4) | 0.180 |
| **20 weeks** |  |  |
| FeFol | Ref |  |
| MMN | 0.14 (-0.59, 0.88) | 0.700 |
| PE | 0.38 (-0.37, 1.1) | 0.326 |
| PE+MMN | 0.48 (-0.29, 1.3) | 0.218 |
| **30 weeks** |  |  |
| FeFol | Ref |  |
| MMN | 0.20 (-0.52, 0.92) | 0.577 |
| PE | 0.39 (-0.35, 1.1) | 0.299 |
| PE+MMN | 0.63 (-0.13, 1.4) | 0.105 |

^a^Mean (SD) gestational age at enrolment was 13.9 (3.3) weeks gestation.

Abbreviations: BMI, body mass index; FeFol, iron folic acid; MMN, multiple micronutrients; PE, protein energy.

**Table A7. Relative risk of LBW and SGA by maternal nutritional supplementation groups**

|  | **LBW** | |  | **SGA** | |
| --- | --- | --- | --- | --- | --- |
| **Supplement** | **RR (95% CI)** | ***p-value*** |  | **RR (95% CI)** | ***p-value*** |
| FeFol | Ref |  |  | Ref |  |
| MMN | 1.1 (0.50, 2.5) | 0.794 |  | 1.0 (0.60, 1.7) | 0.971 |
| PE | 0.98 (0.40, 2.4) | 0.961 |  | 0.71 (0.38, 1.3) | 0.286 |
| PE+MMN | 1.1 (0.46, 2.8) | 0.793 |  | 0.85 (0.46, 1.6) | 0.597 |

Abbreviations: FeFol, iron folic acid; LBW, low birth weight; MMN, multiple micronutrients; PE, protein energy; RR, relative risk; SGA, small-for-gestational-age.

**Table A8. Beta coefficient of the associations between maternal nutritional supplement groups during pregnancy and birth weight**

|  | **Birth weight** | |
| --- | --- | --- |
| **Supplement** | **β (95% CI)** | ***p-value*** |
| FeFol | Ref |  |
| MMN | -2.0 (-10.3, 6.4) | 0.644 |
| PE | 3.5 (-5.1, 12.1) | 0.427 |
| PE+MMN | -0.78 (-9.2, 7.6) | 0.856 |

Abbreviations: FeFol, iron folic acid; MMN, multiple micronutrients; PE, protein energy;
